# Experiences of Students, Teachers, and Parents Participating in an Inclusive, School-Based Informal Engineering Education Program

**DOI:** 10.1007/s10803-021-05230-2

**Published:** 2021-08-19

**Authors:** Yu-Lun Chen, Kavitha Murthi, Wendy Martin, Regan Vidiksis, Ariana Riccio, Kristie Patten

**Affiliations:** 1 Department of Occupational Therapy, New York University, 82 Washington Square East, 6th Floor, New York, NY USA; 2 EDC Center for Children and Technology, 96 Morton Street, 7th Floor, New York, NY USA; 3 Department of Occupational Therapy, Steinhardt School of Culture, Education, and Human Development, New York University, Pless Hall, 82 Washington Square East, 6th Floor, New York, NY 10003 USA

**Keywords:** Adolescents, Youth, STEM, Engineering, Strength-based, School-based intervention

## Abstract

Many youth on the autism spectrum possess interests and strengths for STEM-related postsecondary pathways, yet there are few research-based programs to support those interests and competencies including complex problem solving and social communication. This qualitative study explored the experiences and perceived outcomes of students, teachers, and parents participating in an inclusive, strength-based, extracurricular engineering design program entitled the IDEAS Maker Club. Twenty-six students, 13 parents, and nine teachers in the program completed interviews and program logs while researchers conducted classroom observations over 2 years. Thematic analysis identified five common themes: (1) positive student experience and engagement, (2) skills acquisition, (3) development of interest in STEM and related careers, (4) social relationships and community, and (5) safe spaces that supported self-determination.

## Introduction

Research using nationally representative data reported lower rates of postsecondary education and employment enrollment in students on the autism spectrum^1^ than the general population and those in other disability categories (Shattuck et al., 2012), demonstrating the need to support this population’s career interests and related skills in secondary education. Particularly, youth on the autism spectrum without an intellectual disability may require better support for postsecondary transition, as they were found to be less likely to have daytime employment and educational activities after exiting secondary education than youth on the spectrum with an intellectual disability (Taylor & Seltzer, 2011).

### Youth on the Autism Spectrum and STEM Fields

While youth on the autism spectrum had lower postsecondary education enrollment in general fields (Shattuck et al., 2012), they showed higher enrollment in science, technology, engineering, and mathematics (STEM) than students in the general population students and students with other disabilities (Chen & Weko, 2009; Wei et al., 2013). Youth on the autism spectrum enrolled in STEM majors also showed increased persistence in educational programs than those in non-STEM fields, as indicated by higher likelihoods to persist in a 2-year community college and transfer from a 2-year community college to a 4-year university (Wei et al., 2014). These favorable outcomes may indicate that their potential interests and strengths are conducive to STEM-related careers. Autistic cognitive and perceptual traits, such as highly focused interests, detail-oriented thinking patterns, and distinctive sensory input perceptions, may lend themselves to curricular areas in innovation, problem-solving, and creative thinking (Grandin & Panek, 2013). As engineering is a systematic and iterative approach to designing objects, involving processes and systems, it addresses human needs and real-world problems (Lucas & Hanson, 2016; The National Assessment Governing Board, 2014). This precisely aligns with autistic strengths more than the other disciplines in STEM.

### Barriers to STEM Pathways

Despite youth on the autism spectrum’s high representation in STEM postsecondary education, students on the autism spectrum still face challenges in developing STEM proficiency and related secondary education skills due to their executive function and social communication difficulties (Fleury et al., 2014). Since research-based practices in STEM education that address the learning needs of students on the spectrum are sparse, it affects the teaching effectiveness and teachers’ confidence in teaching STEM subjects to these students (Knight et al., 2013; Smith et al., 2013). A recent systematic review of STEM instruction for students on the spectrum indicated that research-based interventions to primarily support STEM education focus on mathematics and science, with only a few studies of technology instruction and no research on the engineering area (Ehsan et al., 2018). As current educational initiatives in STEM education increasingly emphasize the importance of contemporary engineering education by providing skills necessary for the twenty-first century (Strimel & Grubbs, 2016), it is essential to develop and evaluate autism-inclusive interventions to promote engineering competencies.

The social communication and executive function challenges in students on the spectrum also create barriers to their STEM postsecondary pathways. Executive function is crucial to many required skills for the twenty-first century STEM workplace, such as complex problem solving (i.e., the process of identifying complex problems as well as developing, evaluating, implementing potential solutions) and monitoring (i.e., the ability to assess performance and make improvement or correction; Jang, 2015). Similarly, social communication skills such as coordination (i.e., adjusting actions concerning others), social perceptiveness (i.e., being aware of and understanding others’ reactions), and instructing (teaching others how to do something) are critical components of STEM competencies (Jang, 2015). Hence, interventions intended to support STEM competencies for youth on the autism spectrum need to focus as much on supporting executive function and social-communication skills as academic skills.

### The IDEAS Maker Program

To support students’ STEM competencies, we developed a maker program to be sustainably implemented in public middle schools as an extracurricular program, called Inventing, Designing, and Engineering for All Students, the IDEAS Maker Program (Martin et al., 2019, 2020). Through interdisciplinary collaboration with experts in inclusion, education, Making,^2^ technology, engineering education, co-design, and local schools, we adapted a museum-based maker curriculum to form a school-based informal STEM learning program. The IDEAS Maker Program incorporates inclusive instructional and environmental designs to support all learners in designing, making, and building. Most importantly, it takes a strength-based approach that integrates students’ focused interests in the knowledge and skill of building activities instead of negatively framing autistic interests as deficits. Built upon the conceptual framework of self-determination theory, the IDEAS program seeks to provide semi-structured opportunities for youth on the autism spectrum to see themselves as competent and autonomous actors, relate with peers, and develop essential skills to pursue the postsecondary pathways of their interests (Martin et al., 2020; Ryan & Deci, 2000). Our previous research has shown positive outcomes of the IDEAS program on students’ interests and self-efficacy in technology and engineering, vicarious experience and appreciation of science, and understanding of the engineering design process (Martin et al., 2020).

### The Current Study

This qualitative study aimed to explore (1) the experience and perceptions of students on the spectrum following their participation in the IDEAS Maker Program; (2) teachers’ perceived student outcomes and program impact; and (3) parents’ perceptions and perceived consequences of the IDEAS program following their children’s participation. Students on the autism spectrum’s perspectives and experiences are crucial to providing insight into their learning needs, preferences, and self-perceived program impacts, which are often overlooked in intervention research. In the present study, examining teachers’ perceived program outcomes allowed for a comparison between students’ program engagement and usual classroom participation. Additionally, exploring parents’ perceptions. also provides insight into parental expectations and program impact on students’ daily life.

## Methods

### IDEAS Maker Program Implementation

During the 2018 and 2019 school years, the IDEAS Maker Program was implemented in three public middle schools in a large, urban area in the Northeastern United States that follow a specific autism-inclusion model (Cohen & Hough, 2013; Koenig et al., 2009). All students between grades 6 and 8 in the schools were invited to participate in the IDEAS Maker Program, and a total of 131 students (46 of whom were on the autism spectrum) volunteered to enroll. The maker program was delivered in the format that best fit with each individual school’s club schedule including afterschool, lunch-time, or morning homeroom clubs. Two–three teachers in each school (one subject teacher and one-two special education teacher(s)) facilitated the program. Because the program took place in autism-inclusion schools, all teachers who led the program had already received training in the school district’s autism inclusion model. The teacher training focused on the learning, behavioral, social, and sensory difficulties students on the spectrum commonly experience, as well as classroom strategies and environmental modification to support these challenges (Koenig et al., 2009). For instance, academic support strategies included a daily activity schedule to prepare students on the spectrum for the expected tasks and visual aids to enhance students’ understanding and processing of information. Examples of social support strategies included promoting social opportunities and assigning peer buddies or mentors. In addition, before leading the program, the teachers received 2 days of professional development on Maker principles and activities by museum educators who developed the original Maker program on which the IDEAS Maker Program was based. Teachers mainly facilitated Maker activities and the engineering design process, while students were encouraged to problem-solve with peers rather than seeking teachers’ assistance.

**Fig. 1:**
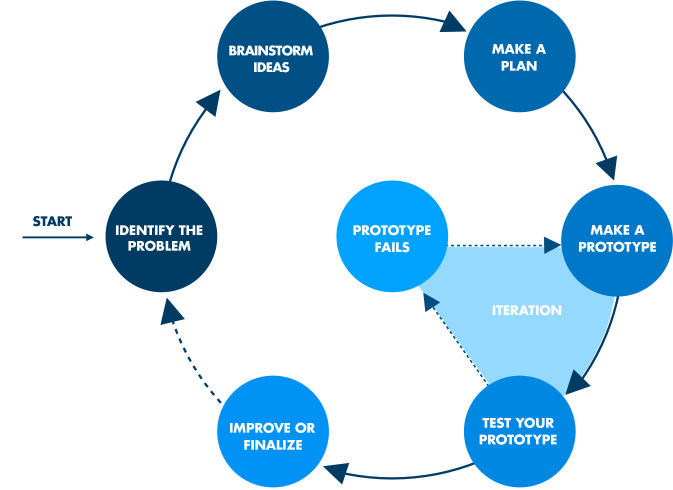
Engineering design process (Martin et al., 2020) (Written permission for print and electronic reuse been obtained)

The IDEAS Maker Program began with 12 Maker activities to build basic making and engineering skills (e.g., engineering design process and 3D printing) and ended with a final project where students incorporate the learned skills into a final product that they design themselves (Martin et al., 2019, 2020). The Appendix provides details on the activities. All program activities encourage students to integrate their interests into making, reflecting a strength-based approach that supports rather than pathologize autistic-focused interests (Dunst et al., 2012; Gunn & Delafield-Butt, 2016). The curriculum utilizes inclusive instructional and environmental designs, such as facilitating learning with multiple modalities using hands-on activities and visual aids and explicit strategy instructions to support problem-solving and monitoring using the engineering design processes (Fleury et al., 2014; Gobbo et al., 2018). The engineering design wheel in Fig. 1 outlined an iterative sequence of six steps for the engineering design process, including problem identification, brainstorming ideas or solutions, making a plan, making a prototype, testing the prototype (and iterating when the prototype fails), and improving or finalizing the design. The process is conceptually parallel to Zelazo et al.’s (1997) problem-solving framework of executive function, which includes four phases (or subfunctions) of executive functioning: problem representation, planning, execution, and evaluation. The engineering design wheel provided students on the autism spectrum with a structured visual guide for identifying goals, planning, problem-solving, and monitoring progress. Along with that, students could engage in a flexible thought process and move from looking closely at one component of the problem to going beyond and seeing the big picture using the EDP. This enabled them to improve their cognitive flexibility and working memory (Bustamante et al., 2018; Householder & Hailey, 2012; Katehi et al., 2009). For example, students could think about their final projects as a whole right from the start of the clubs and then break their projects into smaller components to tackle them but then bring the components together to develop their bigger projects. Also, through constant collaboration and communication with peers and teachers, students learned to persist through their problems and work on solutions together, thus developing self-regulatory skills (DiDonato, 2013). Engineering design wheels were used in the IDEAS Maker Club not only as a visual aid for students to understand the conceptual process but also as a sticker checklist for students to follow through and self-evaluate their progress in engineering design. Lastly, the Maker program was developed in collaboration with a researcher and educator on the autism spectrum chairing the program’s advisory board.

### Participants

Participants included (1) the nine teachers who implemented the program in the three public middle schools over the 2018–2019 and 2019–2020 school years (two teachers from the 1st year left and two new ones joined in the 2nd year); (2) 26 students (17 on the autism spectrum) who participated in the IDEAS Maker Program; and (3) 13 parents of the enrolled students on the autism spectrum. To be enrolled in the schools’ autism inclusion program, all students on the spectrum had an official diagnosis of Autism Spectrum Disorder, an up-to-date evaluation by trained psychologists in the city’s Department of Education, as well as verbal language abilities at or close to age level, intellectual functioning on an average or above-average level, and academic skills on or above grade level. Table 1 lists the demographic data of the student participants. The Institutional Review Board of the research institutions and the school district approved the study, and all participants provided informed consent.

**Table 1:** Demographics of student participants

	Autistic (*n* = 17)	Non-autistic (*n* = 9)
Gender		
Male	14	3
Female	3	6
Grade		
6th	8	4
7th	4	4
8th	5	1
Race/Ethnicity*		
Hispanic	4	1
White	6	1
Black, African American	4	0
Asian	5	7
Pacific Islander	0	0
American Indian	0	0
Other	2	2

*Participants could select more than one ethnicity

### Data Collection

#### Teacher Focus Groups

We conducted semi-structured focus groups with the teachers at both the midpoint (two groups, one per year) and the end of the program (six groups, three per year) to explore their experiences and perceived program outcomes. Two researchers conducted focus groups in the middle of the school year with the whole group at a professional development session to capture teachers’ experiences and student engagement from all of the schools. At the end of the program, teachers at each school engaged in a focus group about their experiences, observed student engagement and outcomes, and positive and negative program characteristics. Teachers were asked to provide case examples to describe student engagement and outcomes. The midpoint focus group lasted 80 min, and the end-of-year focus groups ranged from 35 to 70 min.

#### Student Interviews/Focus Groups

We conducted 17 semi-structured interviews/focus groups with the 26 students (either individually or in a group of three) at the end of the program. These included questions about students’ projects, their impressions of the program, peer interactions, and their interest to rejoin the program in the following year. The interview/focus group questions followed a protocol, but the researchers were flexible and conversational, adjusting according to students’ responses. The lengths of the interviews/focus groups ranged between 4 nad 15 min, depending on the students’ interests.

#### Parent Interviews/Focus Groups

We conducted eight semi-structured interviews/focus groups with 11 parents of students enrolled in the program, including 10 parents of students on the spectrum and one parent of a non-autistic student; six males and five females. Interviews/focus groups were conducted at the end of the program when parents visited maker clubs to view a presentation of final projects, either individually or in a group of one to three. The protocol included questions about parents’ perceptions of the program, perceived student experience or frustration with the program, changes in their children they believed to be associated with the program, and students’ interests at home. The interviews ranged from 3 to 17 min, depending on the parents’ interests and availability.

#### Supplementary Data

##### Teacher Program Implementation Logs

We encouraged all teachers to record a program implementation log to track students’ engagement and response to activities. The implementation logs included structured questions for the teacher to list positive and negative student engagement and feedback to the program. A total of 25 implementation logs were collected across all schools and teachers.

##### Field Observation Notes

We conducted field observations in the program over 2 years, creating 149 observation logs. We conducted exploratory observations during our 1st year, recording details regarding instruction and student engagement. In the 2nd year, we used structured observations focused on teachers' facilitation and students' interests and interactions. Observational data were used to triangulate rather than as the primary source of analysis to focus on participants’ perceptions and avoid researcher bias.

### Qualitative Data Analysis

We recorded all interviews and transcribed them before data analysis. The supplementary program logs and observation notes were analyzed textually. We used thematic analysis based on Braun and Clarke’s (2006) six-phase framework to identify critical patterns across the data. The investigation was both inductive and deductive and included data-driven exploration and coding based on research questions. The first and second authors initially conducted an active and repetitive reading of data, followed by the generation of data- and research question-driven initial codes. The research team discussed and finalized the codes and definitions, and the first and second authors individually coded 15% of the interview data to examine the inter-coder reliability. The two coders achieved a 93% coding agreement and resolved discrepancies in coding through extensive and continued discussions throughout the coding process. Following the coding, we searched for themes presented in the data, identified initial themes, and reviewed the relationships between codes to refine the themes. Data was further triangulated between different sources as a validation strategy. Transparency was maintained through open communication, memo recording, and extensive meetings between team members.

## Results

Thematic analysis identified five themes: (1) student experience and engagement, (2) skills acquisition, (3) interests in STEM and related careers, (4) student relationships and community, and (5) a safe space supporting self-determination.

### Student Experiences and Engagement

Students enjoyed the opportunity and flexibility to make projects that reflected their interests. They were excited and stimulated to develop the skills to use technology that complimented their interests in making, such as 3D printing, TinkerCad (a free computer-aided design software), LED circuits, vibrating motors, and journal making. A student on the spectrum, Robert,^3^ shared that:

My experience has been very, very great. I feel like they teach the basics of 3D printing... and then the fact that you're able to then go on and make your own project is, it's very hands-on. There's a lot of things to make with 3D printing. So creating new designs each time is entertaining.

Creativity was considered another important factor that made the program enjoyable. Students said that the clubs encouraged them to think creatively and pursue their unique ideas through their projects and bring them to fruition. This enabled them to adapt the club experiences to suit their individual making needs. John, a student on the spectrum, expressed his excitement by noting that the club is fun and emphasized that “I like making my creations.” Jerry, another autistic student, further expanded his experience by pointing out that Maker clubs enabled them to work toward a culminating experience, and also engage with and learn from peers.

I liked how we were able to get the ideas built up into one final project. I liked all the other creative ideas that we were able to do to learn about Makers Club...Like [learning about 3D printing, TinkerCAD], all seems to lead up to final projects to learn how we can build something and also I love how we can also socialize with everyone else in the club to get you with some help or just like to talk out.

Non-autistic students also shared the enjoyment and excitement in the program. Asked what she enjoyed about the program, Taylor, a non-autistic student who volunteered to take part in the club for 3 years, talked about enjoying the process of creating and actualizing her ideas, as well as the autonomy and flexibility for making.

Being able to make different things every year and be creative…I really like the journal making and then the 3D printing final project, because you get to have a lot of ideas and put them—test it, see if they work. Adding on to that, you have a lot of freedom when you choose your final project, and of course, it’s nice when it finally comes out. And the process of making the final project is also pretty cool...you have to create your project.

Like the students, parents expressed their appreciation for this program and described how their children experienced happiness in this space because it actively encouraged them to pursue their interests. They felt that the clubs propelled their children to convert their ideas into finished products. A parent of a student on the spectrum reported, “He seems to really enjoy it. He could really get into LEGOs, but he’s enjoying them here.” Another parent underscored the eagerness of their child on the spectrum to participate in each club session as an essential part of their positive experience:

He never dreads that he has to do it or wishes he could come home. It’s been nothing but positive. It’s been great.... he always wants to come here. So I mean that’s the difference I’d see in his excitement outside of our apartment.

Another parent emphasized that task completion gave their child on the spectrum a sense of accomplishment and pride.

When they make something that’s complete, you can really see he’s so happy. And, again, as we know, especially with them, it’s hard for them to complete because they have so many, you know, as we joke, “the creative monkeys” ...so they often can’t stay focused on one thing. He’s like, I did it! I went from A to B. And, of course, with all the help that he, and then he looks at the finished product, and it’s like I can see that that’s something he’s proud of. That, of course, is fantastic—we can tell them that a million times a day. “You can do it if you just focus.” And now he does it and he’s like, “They were right, I could do it.” And that’s really great.

Similarly, a parent of a non-autistic student echoed that her child had a positive experience and great enjoyment from the program.

So in her first years involved in Maker she got really excited, “We’re 3D printing,” and even though I’m an engineer, I’ve never used a 3D printer. I have no idea what a 3D printer does and she was so excited telling me. She told me all the details about it and then last year they built their first robots and she had so much fun with it and we did this and we did that. This year, to her, it was really important to stay involved in it, because I think when she first started there weren’t that many older children involved and she thought it was really important to be there for the younger kids and to help them through things. It’s really exciting to see her grow in that way. She’s talked about it often very fondly and she’s had some really great experiences there.

Teachers mirrored parents’ perspectives regarding their students’ happiness while participating in this program. One teacher acknowledged that students were invested in this program.

He’s just so happy here. He’s constantly building. He’s proud, and he’s genuinely invested in his projects to the point where he thinks about it outside of school. He comes back with new ideas. He wants to work on it outside of school. He’s always excited to show his mom all of his projects.

Teachers’ views resonated with that of students when they spoke about the club being a space that nurtures students’ creativity. Teachers felt that the club activities augmented students’ inventiveness.

Charles is the candidate for this, because he really thinks out of the box. He really brings all that creative ideas and he has a plan in his head, and he will not even think twice. He knows how to carry that plan.

These positive experiences also led students to engage fully in the club’s activities and develop their individual projects outside the clubs too.

The program’s structure and flexible nature augmented students’ creativity, which led them to build on their interests and strengths. During focus group sessions and in their educator logs, the teachers observed that students positively engaged in the activities and club discussions. The teachers’ logs recorded many instances of students’ positive engagement in activities. This matched parents’ observations that their children persisted and continually engaged in club projects even at home.

A parent shared that the program supported the creativity of her child on the spectrum with a structured process to form tangible projects.

He’s learning how to organize it in a form and then put it into a more tangible shape, whether it’s a game or his log I was looking at, of his projects. I mean, it’s still looney, it’s got drawings, but it’s very organized, which obviously is something he has to do for anything he’s going to do. He has to learn how to take his thoughts and put them in a method where he can transmit or communicate with somebody else. And he doesn’t know he’s learning. He’s having fun doing silly things, and that’s important.

### Skill Acquisition

Students developed foundational and content-specific skills about specialized software like TinkerCad, 3D printing, and learning about motors, LEDs, and circuits. Along with this knowledge, the clubs exposed students to independent making, designing, and problem-solving skills by adopting a problem-based learning approach. The engineering design process enabled students to develop and enhance executive functions, which subsequently enhanced their abilities to engage independently in STEM learning. A teacher noted how a program activity facilitated students’ engagement in the engineering design and executive functioning process in the log,

One benefit of implementing this activity was that it fostered problem-solving skills. It is difficult to transfer their design from a paper sketch to a digital design, so students were forced to change something (iteration) to improve their design and make it better. Students were also allowed to take their time and persevere until their name tag was 3D printer ready.

Furthermore, students recognized instances when they solved and used critical thinking to improve their prototypes or make their prototypes from their plans. When Ian, a student on the spectrum, was asked about how he solved a problem regarding LEGO pieces he designed to be 3D printed and put on his Rubik’s Cube, he stated:

They were too thick at first. It took a long time to print. Like a centimeter and half by a centimeter and a half probably. Then I made them thin, but then the pegs at the top of the LEGO piece were too thin. The third time’s a charm because then I got it right because I had to ungroup everything, and it looks cool.

Project failure is a common experience in engineering design, yet students learned to view failure and frustration neutrally or positively, as failing is an expected step in the engineering design process. Sean, a student on the spectrum, described the process of engineering design as iterations of problem-solving and project testing, which shows cognitive flexibility: “we're always testing our prototypes. If it fails, it’s not a big deal because we have plenty of time to try it.” Teachers also recognized students’ progress in overcoming challenges and frustration. A teacher shared an instance when Cameron, a student on the spectrum, experienced a meltdown in the circuit-making activity due to material problems. “So it wasn’t like he couldn’t get it to work. It was like our tape was defective. Nobody’s light lit. Cameron had a total meltdown. [But] he was one of the first ones who figured it out. He made it simple.… it was good practice for him, even though he just melted down like that.” The club appeared to provide a nurturing environment for problem-solving and overcoming challenges.

### Interests in STEM and Related Careers

Some students recognized that the clubs paved the way for their future career interests. A student on the spectrum, John, said, “[an engineer] might be something I want to be when I grow up.” Other student makers also felt that the club provided them with foundational knowledge for a potential career in science and engineering. Sean (on the spectrum) also voiced a similar feeling about the clubs acting as a space where he could develop ideas that could enable him to land a career in engineering. He felt that the clubs blended enjoyment with a purpose and propelled them to think about a future career, “What I’ve enjoyed doing is coming up with a bunch of ideas of what could potentially become a successful … engineering products.” In these instances, the program laid the foundation for students to reflect on their future careers in science and engineering.

### Student Relationships and Community

Students on the spectrum in the program stated that they enjoyed the program’s social environment, which was comfortable and relaxing. When asked what he liked in the program, Sean (on the spectrum) said, “I enjoy being able to hang out with people. I hang out with friends.” Lewis, another student on the spectrum, echoed, “[I enjoy] hanging out with my friends, definitely, especially some that I can’t see during the normal school day.” Since his friends were in different classes, the Maker club allowed him to maintain connections. “At least I still get to see [them] in the Maker’s Club; that’s a good opportunity…I prefer sometimes to socialize and go hang out around and see how people are.” A few parents described their children’s enjoyment of the social aspect of the program: “he likes having that socialization part of it, you know, being like, okay, get to hang out with my friends, and it's not studious.”

Teachers further connected positive student relationships with the program’s emphasis on peer learning and the strength-based, interest-driven environment. Building on students’ strengths and interests, the teachers facilitated student interactions by encouraging peer teaching, which served as initial connections upon which other relationships developed. A teacher shared,

I think one great thing is I just see the relationship building within the groups. A lot of them—like in the beginning, we kind of have to get them to sit together because they might want to sit alone or apart because they're not used to the other person. They don’t know them. But to really foster [student relationships], maybe giving feedback to one another, like using experts who have been here last year and encouraging them to help other students. Because we have one student [on the autism spectrum], Reynaldo, who is like, I’m not a helper. I’m not good at that. But then he’s really good at Tinkercad, so we had him help some other students who were struggling…To see other students learning it from you—I think he enjoyed it...I do think that he starts to build a relationship with that person and then next time, he will approach them.

Two teachers in another school shared an instance where Robert (on the spectrum) developed social relationships based on his strengths and shared interests with peers.

Teacher 1: “One change that we’ve seen in Robert, the first couple of years he was definitely not open to sharing his expertise with other kids and then this year, we’ve asked him to step in so many times and he’s done it–” Teacher 2: “And not only has he stepped in when being asked to, but there’s many times where he just does it on his own...initiates it.” Teacher 1: “something we weren’t seeing before.” Teacher 2: “whether it’s helping a student with Tinkercad or their design, and it could be that a student goes and asks him because they know he knows a lot, and he’s more than happy to show them, and there’s even times where a student is asking for our help, or there’s certain signs that they're struggling and he kind of goes over and just helps them out…And I think that’s happening because this is an area of interest. We were able to tap into something that he’s really passionate about in a really positive way.” Teacher 1: “And then [he] sort of—even being grouped with other kids that share a similar interest, I mean, he’s formed friendships that he probably would not have. I’m talking specifically about some of the [general education] kids that he’s formed that strong bond with. I don’t think that would have happened if it wasn’t for Maker Club.”

Teachers emphasized that the Maker Club fostered a community that appreciated and celebrated autistic strengths and interests, facilitating students’ learning and positive relationships. Opportunities were created where students’ strengths can be seen and valued by their peers. A teacher said, “Most of [the students on the spectrum] are really true makers, and I think that they shine, and I think [their skillset] is really attractive to all the kids.” When asked what impressed them in the program, teachers in a school shared the case of Ethan, a student on the spectrum with a strong interest in Rubik’s cubes:

Ethan, at the beginning of the year, was kind of like hiding his Rubik’s cube under his desk, and he would solve it because he’s obsessed with his Rubik’s cube. And he brought it out during Makers Club, and then everyone was talking about how cool it was, and the kids were interested. And [Teacher 2] and I, we hyped it up too, and that’s what he ended up doing on his final projects both times. And he got our whole homeroom into Rubik’s cubes. It’s a good fidget, and a lot of the kids in his homeroom got fascinated with Rubik’s cubes, and it became his thing. So it became a point where he felt comfortable to bring it out here, and then we promoted that it was awesome. It’s one of your strengths, and then he was able to take it to the classroom, and that’s how he made a lot of friends this year.

### A Safe Space Supporting Self-determination

Students reported enjoying the autonomy in making individualized designs and the sense of reward and self-efficacy when achieving their goals. When asked about their favorite component of the program, students mentioned the opportunity to “create anything you want.” Such freedom and autonomy to create were connected with self-determination, where the students set their goals and strived to achieve them through problem-solving and advocating for assistance. The process of achieving goals also involved self-awareness of one’s abilities and self-monitoring to refine outcomes. When asked whether the program influenced his future goals, Robert (on the spectrum) said, “Well, yes. It helped me think about what I could do on my own and what I could do better. Because making meaning, making something that works is only one step. You could always improve on it.”

This self-determination was associated with confidence and pride in students, parents, and teachers. Charlie, a six-grader on the spectrum, shared that “I think it's a really good experience for me and that I can create many different things, which also can show how people not only with disabilities, but people, how they can be creative and do interesting designs.” A teacher described how being a part of the program was “a badge of honor” for a student on the spectrum, “like I’m a maker, and it just fills his bucket in terms of himself, and who he is and his identity. And to find that identity in this place where he’s very good at it, and he can socialize.”

Both parents and teachers connected students’ self-determination and other positive outcomes with the program’s supportive, strength-based environment. Teachers described the program as a “safe space” for students to overcome challenges and thrive as a community. A teacher recorded in her log: “One benefit of implementing this activity was that students were able to experience some frustration. This facilitated peer-to-peer support and strengthened their advocacy skills. They also practiced problem-solving skills.” Teachers further highlighted the supportive program environment that allows students to show strengths not usually seen in classrooms and to explore common interests and building relationships with their peers.

Although some [activities] are complicated or [all students are] struggling, or they're having a difficult time problem solving, but our students [on the spectrum] find a place where they can thrive, and they can be experts, and they all share a common interest, and they're part of the Maker’s Club, and they're a Maker. It’s an identity that they have, and even with the neurotypical peers, they all share one common interest, and they're all strong in different areas…They're finding other interests that are in common too. And they just have the space to do it. So for me, working as a special educator, that is what I highlight the most when I see them working together and problem-solving and talking in this space…The benefit of having them there is these relationship buildings and for the neurotypical students to see like, oh, we have something in common. Let’s talk to each other. And they say hi to each other in the hallway, so it spreads out.

Similar experiences between students on the spectrum and their non-autistic peers.

## Discussion

This study investigated students, teachers, and parents’ experiences with an extracurricular engineering and design program and their perceived program impact. Interviews, program logs, and field observations suggest that students enjoyed and actively engaged in the program, acquired engineering and problem-solving skills, and developed interests in STEM pathways in several instances. Students valued the program’s autonomy, and their positive program experiences were connected with increased confidence and self-determination. Teachers and parents highlighted students’ growth in social relationships, self-regulation, problem-solving, and self-advocacy, which were associated with an environment that supported students’ interests and strengths and offered opportunities to accomplish self-determined goals and overcome challenges.

This inclusive program was designed to support STEM interests and competencies of students on the spectrum alongside their peers in general education. We interviewed both students on and off the spectrum but focused on the findings of those on the spectrum since students in general education provided similar feedback on the program. Both groups enjoyed their experience and the freedom to create in the program. Students on the spectrum demonstrated progress in skills central for STEM careers including problem-solving, monitoring, and social communication, as well as self-efficacy, autonomy, and self-advocacy skills. The students enjoyed their program experience, and many have applied the skills they acquired during the program outside of the Maker Club, such as creating individual projects at home or exploring careers in engineering and design. They not only acquired technical skills and knowledge but also actively engaged in their interests and strengths in the program, thus cultivating self-determination and developing self-advocacy and collaboration strategies to solve real-world problems.

Key components of the program valued by participants were the support for focused interests, autonomy, self-determined learning, and the inclusive curriculum promoting students’ engagement with peers. The interest-driven curriculum allowed students to flexibly incorporate their interests into program activities and final projects. Participants reported feeling competent through designing and achieving self-determined making plans. These findings aligned with self-determination theory, suggesting that engagement in interest-driven making is intrinsically motivating and associated with increased competence and self-efficacy (Ryan & Deci, 2000). The findings indicate that incorporating focused interests in learning may support autistic motivation, self-determination, and self-efficacy. Promoting self-determination in students on the spectrum is essential as research has identified the association between self-determination and the transition outcomes of students with disabilities (Wehmeyer et al., 2010), as well as the quality of life, employment status, social participation, advocacy, positive identity, and stress management in individuals on the spectrum (Kim, 2019; White et al., 2018). Additionally, the integration of students’ interests supported socialization and relationship building among the students, as shared activities on common interests were necessary social supports for individuals on the spectrum (Muller et al., 2008).

It is important to note possible limitations which impact the interpretation of our results. Participants on the autism spectrum had verbal proficiency and grade-level academic skills, which does not represent the diverse autism spectrum. Future studies should include students with a broader range of verbal and academic skills to explore how the program could support learners on the spectrum with different characteristics. Interviews with students were relatively short, which might not comprehensively capture their experiences. Teachers’ and parents’ subjective opinions might have promoted an idealistic vision, introducing participant bias. The school setting of the study where teachers received extra training to support students on the spectrum might also influence students’ experiences and outcomes. it is recommended that the program be used in conjunction with a strength-based approach that supports and values autistic interests (Cohen & Hough, 2013; Koenig et al., 2009).

Our positive findings led to funding for an expansion of the IDEAS Maker Program to include a wider age range. Thus, we are currently adapting the program to suit both elementary school students (4th grade specifically) and high school students, and we expect the latter can exercise even more autonomy and self-determination using this program model. This future research direction could help students on the spectrum develop STEM interests and competencies at different educational levels.

## Appendix

### IDEAS Maker Activities

**Table:**

Activity	Description	Learning objectives
Activity 1: one sheet of paper	Create a 3D object from a sheet of paper	Students will ∙ Understand and express the difference between 2 and 3D objects ∙ Learn what defines 3D: height, width and depth ∙ Understand that there can be many different outcomes when designing a project ∙ Learn the process of sharing projects and explaining ideas
Activity 2: journal making	Create a handmade journal out of paper using a needle and thread	∙ Design and fabricate their own design journal ∙ Learn bookbinding techniques ∙ Learn and use the basic hand tools to complete the project
Activity 3: intro to 3D printing	Create a solid 3D design out of different materials to simulate 3D printing	∙ Learn what 3D printers are and how they work ∙ Understand how 3D printers print in layers, and how layering happens in complex 3D shapes ∙ Understand glue guns as tools and learn how to safely use them ∙ Learn the properties of different materials ∙ Learn to be conscious of their design choices ∙ Learn the Engineering Design Process (EDP)
Activity 4: wooden blocks	Use wooden blocks to create 3D shapes Design the letters of their name with wooden blocks	∙ Design and fabricate their own design journal ∙ Learn bookbinding techniques ∙ Learn and use the basic hand tools to complete the project
Activity 5: TinkerCAD	Build a 3D Model in TinkerCAD (a computer-aided 3D modeling software)	∙ Understand what 3D CAD software is and how it works ∙ Revisit prototyping and learn about the iteration process ∙ Understand how to transition from physical to digital design ∙ Transfer physical measurements to digital measurements
Activity 6: paper circuits	Design a paper circuit that will turn an LED on and off	∙ Learn the basics of how a circuit works ∙ Understand the basics of what electricity is ∙ Learn what a light-emitting diode (LED) is ∙ Learn what a basic switch is and how it operates
Activity 7: LED greeting cards	Use circuits and LED lights to create an LED Greeting card	∙ Learn how to create multiple circuits in a single project ∙ Gain hands-on experience with simple electrical circuits
Activity 8: motors	Use vibrating motors to create a vibrating object	∙ Learn to use vibrating motors ∙ Learn to create an object from given materials ∙ Review the concept of circuits
Activity 9: final project planning	Come up with an idea for the final project and sketch it out	∙ Learn how to turn their ideas into a visual plan ∙ Decide what their final project is going to be ∙ Create sketches of their final project
Activity 10: prototype take 1	Create the first prototype of their projects out of different materials	∙ Learn the properties of different materials ∙ Learn to be conscious of their design choices and the outcome of those choices ∙ Understand what prototyping means
Activity 11: prototype take 2	Continue building the final project or transfer the prototype to TinkerCAD	∙ Learn to iterate their prototype ∙ Learn to build their project in TinkerCAD
Activity 12: final project board	Create a poster that demonstrates what a student has done in the club	∙ Learn to talk about their design process ∙ Learn to discuss progression through prototypes and iteration ∙ Learn to talk about 3D design and the vocabulary they learned in the program

Details for the activities can be found on this website: https://www.edc.org/ideas-inventing-designing-and-engineering-all-students-maker-program

## Footnotes

1 We use the term “students/youth on the autism spectrum” as it is preferred by the autistic community (Kenny et al., 2016).

2 Making involves ideating, tinkering, testing, iterating and revising creative designs. Maker programs engage participants in the engineering design process of making by constructing products using physical and/or digital tools (Bevan, 2017; Honey & Kanter, 2013).

3 Pseudonyms were used to protect participant confidentiality.
